# Lukewarm Baths to Restore the Waning Power of Age to Youthful Vigor

**Published:** 1891-09

**Authors:** James E. Emerson

**Affiliations:** Bever Falls, Pa.


### Lukewarm Baths to Restore the Warming Power of Age to Youthful Vigor.

BY JAMES E. EMERSON, BEVER FALLS, PA.

I have for a long time (being now past 67) suffered from muscular rheumatism, being feverish from weakness of the muscles. I had for a long time known that old people actually dry up so that the tissues become inactive. I also have known that to use a very stiff scrubbing brush vigorously would toughen the parts and clean the skin ready for absorption through the pores of moisture, so that, by lying in a tepid bath, with the water at from 88° to 92° Fahreheit, for from a half to one hour, the body would actually absorb by weight from two to three pounds of water. Benjamin Franklin, at about 60, began to feel greatly the encroachments of old age, so he went to Dr. Darwin for advice. The Doctor recommended to him the lukewarm bath, to be taken twice a week. Franklin followed this advice, and very soon noted the beneficial effects of these warm baths upon his aged body. He is said to have continued their use up to within a short time of his death, which was at 84, and to the very last was strong and vigorous in body and mind. It restores elasticity and smoothness to the skin; it loosens the tissues and thereby brings back fullness and soundness of limbs. It prevents eruptions of the skin, and presently it removes them often even from the face. It prevents the body giving off too much heat, which enhances nutrition.

